# “I guess it looks worse to me, it doesn’t look like there’s been a problem solved but obviously there is”: a qualitative exploration of children’s and their parents’ views of silver diamine fluoride for the management of carious lesions in children

**DOI:** 10.1186/s12903-021-01730-w

**Published:** 2021-07-23

**Authors:** N. Seifo, H. Cassie, J. R. Radford, N. P. T. Innes

**Affiliations:** 1 School of Dentistry, University of Dundee, Park Place, Dundee, DD1 4HR UK; 2 School of Dentistry, Cardiff University, Heath Park, Cardiff, CF14 4XY UK

**Keywords:** Silver diamine fluoride, Caries, Acceptability, Parents, Children

## Abstract

**Background:**

Despite growing evidence to support the use of silver diamine fluoride (SDF) for managing carious lesions, and the increased interest in SDF worldwide, uptake in the UK remains limited. This study explored parents’ and children’s views and acceptability of SDF for the management of carious lesions in children.

**Methods:**

Eleven semi-structured face-to-face interviews were conducted with 11 parent–child dyads recruited from patients attending Dundee Dental Hospital and School. Interviews were transcribed verbatim, coded and thematically analysed.

**Results:**

Previous dental experience varied across all child participants. Of the 11 children, five had undergone general anaesthesia (GA) for multiple primary tooth extractions. Two had received SDF treatment. Child participants expressed concerns about being picked on by their peers, if they had discoloured anterior teeth. Younger children appeared less concerned about the discolouration and child’s gender did not appear to influence parents’ decision-making, nor the child’s preferences regarding the use of SDF. Parents considered SDF to be particularly useful for anxious or uncooperative children but raised concerns about potential bullying at schools due to the unacceptable dental aesthetics when SDF is applied to anterior teeth. They believed they may be judged by others as neglecting their child’s oral health due to the black staining. Both parents and children were more accepting of the SDF when applied to less-visible posterior teeth. Parents accepted the use of SDF if such treatment avoided extractions under GA.

**Conclusion:**

Despite the unfavourable aesthetics of SDF (black staining), parents appreciated SDF treatment, especially for uncooperative or younger children. However, both parents and children shared concerns about bullying at schools as a consequence of the black staining. Raising awareness about SDF was identified as one approach to encourage the uptake of SDF.

**Supplementary Information:**

The online version contains supplementary material available at 10.1186/s12903-021-01730-w.

## Background

Silver diamine fluoride (SDF) is a clear odourless liquid that was first investigated for managing carious lesions in Japan in 1969 [1]. However, since it was cleared by the United States Food and Drug Administration in 2014, there has been increasing interest in its use [2]. More recently, the use of SDF as during the Covid-19 pandemic has been highlighted as a treatment option, due to it being a non-aerosol-generating procedure for managing carious lesions [3].

SDF contains silver and fluoride, which act synergistically to arrest carious lesions through a variety of mechanisms [4]. The silver ions can constrain bacterial growth by interacting with bacterial cell walls and enzymes, and impede dentine collagen degradation. The fluoride ions promote remineralisation by forming fluorohydroxyapatite and inhibiting matrix metalloproteinase activities and therefore dentine collagen degradation [5].

There is a growing body of evidence supporting the use of SDF for managing carious lesions in children [6]. However, one side effect is that the carious lesion is stained black. It has been suggested that this staining could be a barrier to its use for some parents, especially for anterior teeth [7]. Yet, there is other evidence to suggest that parents may view this discoloration as a positive sign that the treatment has been effective [2]. A survey-based study in the United States found that staining on posterior teeth was more acceptable than staining on anterior teeth. In addition, although staining on anterior teeth was undesirable, most parents preferred SDF as a treatment option over techniques that required the use of sedation or general anaesthesia (GA) [8].

It is not clear yet where the threshold for parents to accept use of SDF lies, particularly with the undesirable effect of tooth staining, or whether there are other barriers or enablers for its use. Furthermore, children’s views of SDF have not yet been investigated. This is despite an increasing emphasis on capturing children’s views within health services research to ensure that the treatments they are offered and their views on treatment outcomes are heard and addressed [9]. Exploring parents’ and children’s preferences towards the use of SDF, will address an evidence gap, support decision-making and treatment planning, and could contribute to strategies to increase use of SDF in dental practice.

This qualitative study explored parents’ and children’s views on the acceptability of SDF for the management of dental carious lesions in children and taking into consideration the child’s previous dental experience.

## Methods

### Study design

Semi-structured face-to-face audio recorded interviews with parents and children were carried out between August 2019 and January 2020. The consolidated criteria for reporting qualitative research (COREQ) [10] was used to ensure quality.

### Participants and recruitment

Parents and their children (aged 4–12 years old), attending the Child Dental Health Clinic at Dundee Dental Hospital and School (DDH&S) were eligible. To ensure sample heterogeneity (for children’s ages and gender), a purposive recruitment strategy was adopted [11].

Potentially suitable participants were identified by clinicians in the Child Dental Health Clinic and informed about the study. If they were interested in participating, the lead researcher (NS) explained the study in more detail and gave the parents a study information pack with a Parent Information Sheet and a Child Information Sheet, customised for that age group. The information pack also included a reply slip and Freepost envelope to confirm their participation, contact details and the best time for them to be contacted.

Participants returned the reply slip by putting it in a designated box at the clinic reception at their next visit to DDH&S or by using the freepost envelope provided.

### Consent and ethical review

All methods were carried out in accordance with relevant guidelines and regulations. Prior to the interview, the study aims were discussed further with the participants. If they were willing to participate, informed consent was obtained from parents and/or legal guardian of participating children. The child assent process involved speaking to and explaining the study to the child in simple language. After the interview, each child was given a £10 voucher as a token of thanks for participating.

This study was approved by the Research and Development Management Department at National Health Service (NHS) Tayside (IRAS ID: 254563, REC Ref: 19/ES/0042). Caldicott approval was obtained from NHS Tayside to allow access to personal data of potential participants for recruitment (Ref: IGTCAL6259).

### Data collection

Semi-structured face-to-face interviews were conducted by NS in a non-clinical meeting room within DDH&S. An open-ended question and ‘probing approach’ were undertaken. The parent was interviewed first, then the child, with both being present in the room for each interview.

An interview schedule was developed to examine acceptability of SDF from a parent and child perspective, using age-appropriate language (Additional file 1). This interview schedule was informed by the literature as well as previous work conducted by the authors, exploring Dental Care Professionals’ acceptability of SDF [7]. Using clinical photographs of patients before and after SDF treatment (Additional file 2), questions were focused on the acceptability of SDF, together with factors that influence decision-making regarding other treatment options.

The interview schedule was piloted with two parent–child dyads prior to starting the study. No revisions were made. Data from these pilot interviews were not included in the analysis. Data collection was carried out until saturation was reached i.e. when no new themes, categories or explanations were emerging.

### Data handling and analysis

All identifiable data were anonymised, audio recordings were securely transferred to a professional transcription service and transcribed verbatim. For the analysis, participant names were pseudonymised in the transcripts.

Transcript accuracy was checked prior to analysis by one researcher (NS). Data were managed using NVivo 12 software QSR (International Pty Ltd., Melbourne, Australia). Data were organised and classified according to key issues, concepts and emerging themes. This was carried out using a thematic analysis and framework approach [12].

To minimise bias and ensure consistency, NS and HC (an experienced qualitative researcher) double coded a sample of transcripts independently and in duplicate. The coding framework was developed following an initial review of three transcripts. This was then assessed by one of the researchers (HC) who had not been involved in conducting the interviews. Development of the codebook was an iterative process with adaptations made through discussion.

## Results

Eleven interviews were conducted, each with one of 11 parent/child dyads. The 11 parent participants comprised three fathers, seven mothers and one grandmother. All children were regular dental attenders and of the 11 children, 55% were boys. The age of child participants ranged from six-year-olds or younger (n = 4), seven to nine year-olds (n = 3) and over nine years-olds (n = 4). Interviews were from 15 to 25 min in length.

### Children’s previous dental experience

The previous dental experience of children varied, but all of those interviewed had received dental oral health assessments (“check-ups”). For two of the children, the only intervention had been the placement of fissure sealants. Five children had undergone GA for multiple primary tooth extractions. One had received dental extractions with local analgesia. Three children had received restorations, one of whom had endodontic treatment for a permanent tooth. Three had received a crown placed using the Hall Technique (HT) and two children had previously received SDF treatment at DDH&S.

Parents whose children had undergone multiple tooth extractions under GA described the experience as traumatic for both themselves and their child. In addition, they suggested that they felt that an excessive number of teeth had been extracted during the procedure. Children who could recall their GA experience, reported it to be very distressing.

*“I was angry, I was angry, I was angry, ‘cause he, he was sitting there crying for mum and dad and we were there and there’s nothing I could’ve done er, he didn’t want put to sleep. The, the nurses, give the nurses their due, they tried everything, give him a gas until he fell asleep. It’s when he woke up was when the pain kicked in, and to see a child going through a lot of pain after this being done, getting them all taken out”*

**(Parent I, father to a 10 year-old boy)**

Parents of children who received a HT crown, reported satisfaction with this treatment approach, despite it being slightly uncomfortable for the child. However, they did not consider the HT to be a straightforward procedure as it was time-consuming to choose the crown dimension and to carry out the placement of the crown. Children who had been treated with the HT suggested that having the crown fitted was acceptable though they experienced some discomfort.

*“…so I went and got the crown. It didn’t really hurt, it only hurt, like, a tiny bit because he really hard pushed on my tooth to stick it on, but it never really hurt”*

**(Child E, girl aged 9 years old)**

### Parents’ views of SDF

The two following overarching themes emerged from the interviews with parents: ‘perceptions of SDF’ and ‘factors influencing decision-making’ (Table 1).

**Table 1:** Themes emerging from the interviews with parents and children

	Themes	Sub themes	Topics within the theme
Interviews with parents	Perceptions of SDF	Perceived advantages of SDF	Minimal child’s cooperation required Non-invasive stress-free treatment The possibility of saving the tooth from extraction Promoting good oral health
		Aesthetics	The black staining of arrested lesions Minimising the staining to improve acceptability
	Factors influencing decision making	Perceptions of others	Bullying at schools or nurseries Other people’s judgment
		Relative visibility of the tooth	Anterior or posterior tooth
		Self-consciousness	Child’s age/gender’s impact on parent’s decision
		Longevity of tooth	Conflicting views on when SDF was most advantageous
		Relative merits of alternative approaches	Saving the tooth from extraction Avoiding GA Choosing between SDF and HT
		Preferences and recommendations of others involved in treatment	Child’s preferences Dentist’s recommendations
		Financial considerations	The cost to the NHS
		Child tolerance of treatment	Child’s sensitivity to strange smells or tastes
Interviews with children	Child’s acceptability of SDF	Relative visibility of the tooth	Anterior or posterior tooth Size of the carious lesion
		Peers’ perceptions	Fears of bullying by peers at nurseries or schools
		Previous experience	Impact of previous dental treatment experience (positive and negative) on child’s treatment preferences

### Perceptions of SDF

While many acknowledged the advantages, they also identified disadvantages. The two sub-themes that emerged were ‘perceived advantages of SDF’ and ‘aesthetics’.

#### Perceived advantages of SDF

Some parents believed that SDF treatment could be particularly useful for children where their anxiety or inability to co-operate with or tolerate some treatments may limit other interventions. Parents perceived SDF to be a non-invasive procedure that children would not find stressful and moreover beneficial to introduce children to the dental environment.

*“I think it’s a great treatment for kids, especially young kids that are apprehensive about coming to the dentist or the dentist sort of, er, looking in their mouth and things like that”*

**(Parent J, father to a 5 year-old boy)**

Parents felt further advantages were the delay or avoidance of treatment under GA and in promoting good oral health.

*“it made a massive difference to Jack when he got that put on. He was kind of scared to brush his teeth because he was in that much pain, and then after that product was put on, he could brush his teeth. It helped him help his other teeth that were going to be staying”*

**(Parent G, father to a 10 year-old boy)**

#### Aesthetics

There were concerns about lesion staining, especially of anterior teeth. It was stated that an SDF treated tooth could look worse than the original untreated carious tooth.

*“I guess it looks worse to me, it doesn’t look like there’s been a problem solved but obviously there is”*

**(Parent B, mother to a 5 year-old boy)**

Parents suggested that developing a way to minimise the black staining would improve SDF acceptability.

### Factors influencing decision-making

Parents did not appear to have a clear opinion about choosing SDF for their child. There were many factors influencing decision-making. The sub-themes identified within this theme were ‘perception of others’, relative visibility of the tooth’, ‘self-consciousness’, ‘longevity of the tooth’, ‘relative merits of alternative approaches’, ‘preferences and recommendations of others involved in treatment’, ‘financial considerations’ and ‘child tolerance’.

#### Perceptions of others

Some parents believed that the discolouration, especially of anterior teeth could result in the child feeling uncomfortable or anxious when they spoke or smiled and may make them a focus for bullying. Of note, the school culture seemed to be influential in that if the school reported endemic problems with bullying, the parents were more hesitant to consent to SDF treatment for their child.

*“then the next thing, a kid’s at school with black teeth… I think I’m all about the anti-bullying, and this to me would lead to bullying”*

**(Parent E, grandmother to a 9 year-old girl)**

Black-stained teeth were associated with drug abuse in adults and there was concern this may be reflected towards children too. Fear of what ‘others would think’ and a feeling that parents may be judged by others as neglecting their child’s health if their child had black-stained teeth. This was also because they thought a blackened tooth appeared similar to an untreated carious lesion and people may not be able to differentiate. Parents believed they would rather have the teeth extracted as they would be less likely to be judged by others, with people assuming the teeth had exfoliated earlier than normal.

Parents believed SDF could be a more acceptable if people had greater awareness of it. With greater awareness there may be less chance of being judged by others and therefore, parents would be less apprehensive about choosing SDF for their child.

*“Maybe more to the future, once it’s been around a while, people know more about it, they’d maybe understand what it was and they maybe wouldn’t judge so much, you know?”*

**(Parent H, mother to a 7 year-old boy)**

#### Relative visibility of the tooth

The SDF-treated carious lesion’s visibility seemed to be the most influential factor on parents’ decision and more acceptable on their child’s posterior teeth since it would not be as visible. Some parents commented that the arrested carious lesions may not look any worse than amalgam fillings:

*“if it is in a back tooth, a back molar, then it’s the equivalent of one of the old iron or dark fillings”*

**Parent D, mother to a 6 year-old girl**

The staining caused by SDF on anterior teeth was unacceptable for many parents.

*“Hmm, it looks awful! It looks awful. …. certainly on a front tooth, I wouldn’t want that on my child”*

**Parent F, mother to a 10 year-old boy**

However, the size of the lesion was of importance with SDF a possible option if the lesion was relatively small and not very noticeable.

Some parents said that they would not mind the appearance if SDF would stop the lesion progressing and avoid any further intervention.

#### Self-consciousness

Younger children were considered less self-conscious than older children and may not mind the staining, therefore SDF-related discolouration may be less of a barrier for them. But with older children, parents were more concerned with the possibility of bullying.

*“It wouldn’t have bothered me before, now that he is at school, it would worry me that other children might pick up on that and that might be an issue, only because of children’s behaviour. Yeah”*

**(Parent B, mother to a 5 year-old boy)**

Gender did not appear to influence parents’ decision-making regarding the use of SDF for their child.

#### Longevity of the tooth

Parents had conflicting opinions about how the length of time until the tooth was expected to exfoliate might affect their decision. Some thought that if the teeth were to be lost within a short period of time i.e. less than six months, they would consider SDF treatment. Conversely, some believed that if the tooth to receive the SDF treatment would fall out in few months, they would rather just take the tooth out and if the tooth was likely to last longer, they would opt for SDF.

*“If she was on the crust of her new teeth coming through and it would only be, like, two or three months, I would say, “Och, yeah, take them out then.” What’s the problem? Young kids at that age do lose their teeth anyway. But if it was going to be a longer period of time, six months plus without teeth, I would say, “Nah, get this treatment done”*

**Parent E, grandmother to a 9 year-old girl**

#### Relative merits of alternative approaches

Parents took alternative treatment options to SDF into consideration. Some parents who were less accepting of SDF showed more flexibility if SDF was the last resort that could save the anterior teeth from extraction believing that a black-stained tooth was better than not having the tooth at all.

Some parents would choose SDF, albeit hesitantly, if it avoided the child undergoing GA because of its associated risks. Even if the other option was treatment under inhalation sedation, parents tended to prefer SDF.

*“Mhm. I wouldn’t want her put to sleep for her teeth to be filled or treated. I’d rather that she had that, the SDF because there’s such a risk with general anaesthetic. Well, not a massive risk but there’s still a risk with GAs isn’t there”*

**Parent A, mother to a 5 year-old girl**

In contrast, several parents insisted that they would never choose SDF for their child’s anterior teeth and considered the outcome unacceptable with extractions more acceptable than a visible, black-stained tooth.

*“Yeah, that’s awful. I would rather he got put to sleep and them taken out, yeah. I would rather not have them*”

**Parent H, mother to a 7 year-old boy**

Parents were asked about their preferences between HT or SDF for their child, since both techniques share some clinical indications. Some preferred the SDF option because aesthetically, the crowns were silver, cover the whole tooth and also not very aesthetic whereas SDF only affected part of the tooth. Furthermore, they thought applying SDF was simpler and more acceptable for the child.

*“If I remember rightly it was a little bit uncomfortable when they were pushing it on, trying to fit it, so I mean, this would be a lot simpler. You know, the back teeth, getting that stuff on, it would probably be a better option”*

**Parent H, mother to a 7 year-old boy**

#### Preferences and recommendations of others involved in treatment

Some parents took their child’s treatment preferences into account not wishing to force their child to receive treatment.

*“I think as a, as a parent yeah, I mean it would obviously depend on… because it’s work to be done to the child, so I would want to have their opinion on it, and I would never force something”*

**Parent J, mother to a 9 year-old girl**

Others suggested that the dentist was the expert and they reported having full trust in them. They were happy to choose whatever treatment the dentist believed to be the best option for the child.

#### Financial considerations

Some parents also considered the cost of treatment to the NHS. If there were two management options with similar success rates, they would prefer the more cost-effective treatment approach.

*“Um, and also I am interested in what it costs um, the NHS and, and things like that because that’s something I think that we do need to be responsible citizens and if there are treatment options that are going to be more cost effective for the NHS then I do think um, that it’s, that it’s our duty to consider those”*

**Parent J, mother to a 9 year-old girl**

#### Child tolerance

Parents suggested that some children with sensitivity issues towards new or strong smells or tastes may not tolerate SDF because of the taste.

*“The only thing he has a problem with, he’s got, like, sensory things, you don’t like tastes and smells and things. So, if it’s certain varnishes and the coatings and things that they’re using, if they taste funny or smell funny, he’s like, “No!” He’s more frightened of that than anything else”*

**(Parent H, mother to a 7 year-old boy)**

### Children’s views of SDF

Younger children were shy and generally less talkative than older children, especially at the beginning of the interview. They tended to be more responsive to yes and no questions than open questions probing for more expansive responses. Children were shown pictures of SDF treated teeth as part of the interview and they described them as “rotten”, “weird”, “silly”, “ugly” or “disgusting”. One overarching theme, ‘child’s acceptability of SDF’ emerged from the interviews with children.

### Child’s acceptability of SDF

Factors influencing children’s views could be categorised into three sub-themes: ‘relative visibility of the tooth’, ‘peers’ perception’ and ‘previous experience’ (Table 1).

#### Relative visibility of the tooth

When asked how they felt about having similar treatments on their teeth, some children seemed more accepting of black staining on their posterior teeth, believing that others would not see it.

*“Um, on the back, that’s okay, kind of. I don’t mind to have [it] because people wouldn’t really, like, see it when, like, um, like, when I’m like, talking or anything, because it’s in my, like, one of my back teeth, so they wouldn’t really see it”*

**(Child E, girl aged 9 years old)**

However, most children were not keen on SDF staining being visible on their anterior teeth as they thought it looked like a rotten tooth.

Similar to parents, if the lesion was fairly small however, there was less opposition.

*“Um, if they were at the front, I wouldn’t really like it. If it was just a little at the front, then that would be okay, like that one”*

**(Child F, boy aged 10 years old)**

#### Peers’ perceptions

Children worried others would comment on their appearance and they may be picked upon by their peers. One child who had previously suffered from bullying at school commented:

*“Oh, the front teeth, no, no ….. Absolutely not because they look not that nice. I wouldn’t like that because it will look silly, because I think I’ll get bullied. And then people will just go, like, “Amy, what are your teeth like? They look ugly.” I think they’ll say that”*

**(Child E, girl aged 9 years old)**

Some children reported that they would be unwilling to accept SDF treatment, preferring to have their teeth extracted. The children’s responses indicated that older children were more aware of the staining and how that could lead to being picked on, than younger children. It was suggested that younger children may be less self-conscious or worried about the implication or reaction from others of having black staining of their teeth.

*“If they see them and they think it’s rotten then I think they’d possibly laugh if they’re in like the older classes, but otherwise if it was friends they would try and support them”*

**(Child J, girl aged 9 years old)**

Overall, boys and girls did not appear to have different opinions about having their teeth treated with SDF, with both reporting similar perceptions.

#### Previous experience

Previous dental experience appeared to influence children’s opinions in relation to future dental treatment. Most of the children interviewed had experienced multiple tooth extractions under GA and described the experience as very distressful. They stated that they would choose SDF treatment if it could avoid a further GA.

*“Yes, I would prefer that one. Yeah, ‘cause getting all those teeth pulled out I couldn’t go through all that pain again, oh, that was so sore”*

**(Child I, boy aged 10 years old)**

Conversely, one child appeared less concerned about undergoing GA and reported that they would prefer a GA again rather than SDF treatment. This child participant had also previously had crowns fitted with the Hall Technique applied and said they would prefer this to SDF. A possible explanation is fear of SDF as unknown.

## Discussion

This is the first study to explore, in depth, both parents’ and children’s acceptance of SDF. Previous studies have explored parents’ views on the acceptability of SDF [8, 13–15]. Furthermore, this study captured younger children’s views (as young as four-years-old), an age group often overlooked in research. We found parents’ and children shared similar views as to the acceptability of SDF; both expressed concerns about SDF-induced black staining on anterior teeth, but tended to be more accepting of SDF treatment on smaller lesions and the less visible posterior teeth. Older children seemed more concerned about the discolouration. Of note, there was no difference between boys’ and girls’ perspectives. When considering treatment options for their child’s carious lesions, parents expressed diverse preferences with seemingly conflicting influences. For example, there were varying views about whether teeth due to exfoliate soon influenced decision-making positively or negatively towards the use of SDF.

The most commonly reported advantage of SDF treatment related to the benefits of treating uncooperative children. It was agreed that this non-invasive treatment should limit distress to the child and, therefore, could be useful in acclimatising children to the dental environment, and encouraging them to accept more complex procedures in the future.

Parents whose children had required GA in the past, stressed the benefits of SDF in avoiding or delaying GA. They suggested that if SDF had been an option for them previously they would have chosen it, despite the discolouration because, similar to findings from other studies, the GA experience was notably traumatic for children and their parents [16, 17]. A qualitative study conducted exploring parents’ experience of their child’s dental GA found that some parents struggled to accept the use of GA although others believed it was superior to conventional treatment. Nonetheless, all parents reported some level of anxiety, fear or worry associated with their child undergoing dental treatment under GA [18].

Children who had undergone a dental GA also reported finding the experience unpleasant. They were troubled by being put to sleep and the pain experienced after the procedure. This was also found by Rodd et al. [19] when exploring children’s views of having GA dental extractions. Children felt scared and worried before their admission. Using a different methodology, Baghdadi et al. [20] used children's drawing as a projective measure to understand their experiences of dental treatment under GA. Again, it was the unknown that provoked stress although the anaesthetic gas mask also caused distress.

Most parents and children expressed concerns about the aesthetics of dark staining as a result of SDF, especially if this was visible on anterior teeth. They also worried that it might increase children being bullied or picked on at school or nursery. Older children i.e. seven years or older seemed more concerned and aware of this. It is a feasible concern, given that physical appearance is the most frequently cited reason for bullying. Globally, 15.3% of students who have been bullied, reported being made fun of, because of how their face or body looks [21].

In a similar vein, parents also feared being judged by others if their child had black discoloured teeth since others may think that they have been neglecting their child’s oral health and black teeth are associated with drug abuse [22]. A few parents reported that they would prefer their child’s teeth to be extracted over having black stained SDF treated teeth. These concerns are not limited to SDF treatment. Maguire et al. [23] reported that parents raised similar concerns about crowns in their child’s mouth being a visible sign of failure in their parental responsibilities. Parents suggested this barrier could be overcome through raised awareness of SDF as a treatment and understanding that a well looked after tooth does not necessarily mean a white tooth.

Greater parental acceptance of SDF for posterior teeth than for anterior teeth is supported by the findings of previous studies exploring parents’ perceptions of SDF [8, 24]. We found here that children also expressed less opposition to SDF on posterior teeth compared with anterior teeth.

Parents also reported that their child’s age was a consideration, as older children may be more self-conscious and less likely to accept the treatment. It was suggested that younger children may be less self-conscious than older children and may not mind the discolouration as much. In contrast to this, a study exploring perceptions around dental aesthetics found that younger children (aged 2–7 years-old) have the perception that beautiful teeth are shaped and white, while ugly teeth are shapeless and have cavities in them [25]. Furthermore, children aged six years-old were capable of appreciating the aesthetics of anterior tooth restorations [26].

Although it was suggested that the gender of the child may have affected parents’ views around aesthetics previously, there was a feeling that boys and girls are now treated more similarly. The child interviews identified no discernible difference between boys’ and girls’ perceptions, with both sharing similar beliefs about SDF treatment. This finding is supported by a study which explored body image perception. In this study the results suggested that boys’ and girls’ body image perceptions show similar trajectories [27].

It should be noted that some of the themes to emerge from the interview data; perceptions of others, visibility and self-consciousness are very much interlinked, and focus on the overarching theme of aesthetic outcomes specific to SDF treatment. Whereas other themes, such as communication, financial considerations and child’s tolerance for treatment were standalone and probably applicable to other dental treatments in addition to SDF.

Socioeconomic factors, educational level and residence location (urban, suburban, or rural) were not explored in this study. Therefore, it was not possible to assess whether these variables impacted on the level of parental acceptance of SDF. Although participants were recruited only at Dundee Dental Hospital and School, patients’ values and expectations are unlikely to vary considerably across the whole population. Although the analysis of the results of the qualitative component relied solely on the lead researcher, to minimise bias, a sample of interview transcripts were double coded independently by them and another investigator who was experienced in this research discipline.

Even though there is large body of dental research pertaining to children, this has been *about* them rather than involving them directly [28]. This may be due to the flawed belief that data obtained from children may be unreliable and invalid [29]. However, children can be credible participants and experts on their own lives, providing invaluable knowledge and a unique perception [30]. Children's involvement in dental research has not only been embraced during the last decade but enriched the knowledge base in order to improve their well-being [9].

## Conclusions

The overarching themes from interviews with parents were (1) their “perceptions of SDF” with associated sub-themes of “SDF’s perceived advantages” and “aesthetics” and (2) decision-making” for their children’s treatment with emerging sub-themes of; perception of others, visibility, self-consciousness, duration, alternative approaches, communication, financial considerations and child tolerance. The single overarching theme from the interviews with children was the “acceptability of SDF” to them, and could be categorised into three sub-themes: visibility, peers’ perception and previous experience.

Children and parents shared similar views particularly around aesthetics. SDF-induced black staining on anterior teeth was perceived as possibly leading to children and parents being judged negatively by others for their care and to bullying of children. Consequently, SDF treatment was more acceptable on posterior teeth. Younger children seemed less concerned about discolouration. The child’s gender did not appear to influence parents’ decisions or the child’s preferences for use of SDF.

Parents believed that SDF would be particularly useful for anxious and uncooperative children and the procedure’s simplicity could make SDF an entry point to more complex procedures. Parents appeared to consider different factors before choosing SDF or not for their child. Their often conflicting viewpoints were associated with specific situations. This points to the importance of clinicians understanding the individual circumstances, previous experiences, preferences and anxieties for each child and their parents in shared decision making and treatment planning.

## Supplementary Information

**Additional file 1**. Topic guide.

**Additional file 2**. Photographs shown to parents and children as part of the interviews.

## Data Availability

The datasets used and analysed during the current study and a description of the coding tree are available from the corresponding author on reasonable request.

## Abbreviations

SDF: Silver diamine fluoride
DDH&S: Dundee Dental Hospital and School
NHS: National Health Service
GA: General anaesthesia
HT: Hall technique
