# Supplementary material for: “I guess it looks worse to me, it doesn’t look like there’s been a problem solved but obviously there is”: a qualitative exploration of children’s and their parents’ views of silver diamine fluoride for the management of carious lesions in children
Source: BMC Oral Health. 2021 Jul 23;21:367. doi: 10.1186/s12903-021-01730-w (PMC8298692; doi:10.1186/s12903-021-01730-w)
Supplement: Supplementary file 1 — Additional file 1. Topic guide. [file 12903_2021_1730_MOESM1_ESM.pdf]

Topic Guide

Study title: Parents/carers' and children's views of silver diamine fluoride for the management of carious lesions in children: a multi-methods study

Parents/carers interviews

Introduction

My name is Nassar Seifo, I am a trained Paediatric Dentist and doing a PhD at Dundee Dental School and Hospital. Thank you for coming and agreeing to participate in this study.

This study will basically explore what parent and children think of a type of fluoride. This fluoride agent that can actually stop the decay after it has started, but it stains the decayed bit of the tooth black permanently.

Let's go through the consent form together and you can tick the boxes if you agree please?
Can we go through the assent form with your child?
Do you have any questions before we start?
Are you ok with me turning on the audio recorder now?

Let's move on the to the first part of this interview which will be about your child's previous **DENTAL EXPERIENCE**

- Has your child been to the dentist before? If yes, what for?
- What did the dentist do?
- How often do you take your child to the dentist?
- How does your child feel about going to the dentist?
- Does anything concern you? if yes, what concerned you the most when your child was in the dental chair and why? If no, why?
- How was the overall experience at the dentist? Why?
- Has your child had any complications after the treatment? If yes, what?
- How would you feel about taking your child to the dentist again for similar treatment?

Moving on to the second part which explores your thoughts on **SILVER DIAMINE FLUORIDE**

- Here are photos of decayed teeth, what do think of them?
- Here are the same teeth after the dentist applied the fluoride liquid that stains the teeth but stops the decay, what do you think of them?
- How would you feel if your child received a treatment that would stain the tooth but prevent any pain or infection in the future, without local anaesthetic?
- Imagine that your child had a decayed milk tooth that will cause pain if left untreated. If the tooth was a back one, which of the following treatments you will choose? Why? (present photos of different treatments)
- What if the tooth was a front one, would it change your choice? why?
- If your child could not cope with treatment and the only option was to do treatment under sedation with inhalation (laughing) gas or avoid the inhalation gas by applying SDF, which option would you choose? Why?
- If your child could not cope with treatment and the only options were to extract the tooth with your child under general anaesthetic at hospital or avoid your child having a general anaesthetic by applying SDF, which option would you choose? Why?
- If you decided to choose SDF treatment, how do you feel about the need for this SDF to be re-applied every 6 months at the dentist?

Children interviews

- Can you remember going to the dentist?
- Can you remember why you went there?
- What did the dentist do?
- How did you feel there?
- Would it bother you to have your front/back tooth like this one (present SDF cases), why?
