# Supplementary material for: “I guess it looks worse to me, it doesn’t look like there’s been a problem solved but obviously there is”: a qualitative exploration of children’s and their parents’ views of silver diamine fluoride for the management of carious lesions in children
Source: BMC Oral Health. 2021 Jul 23;21:367. doi: 10.1186/s12903-021-01730-w (PMC8298692; doi:10.1186/s12903-021-01730-w)

University of Dundee

Photographs shown to parents and children as part of the interviews

Before SDF treatment

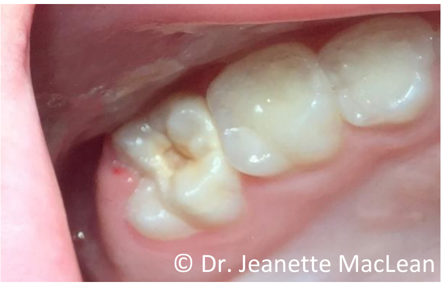

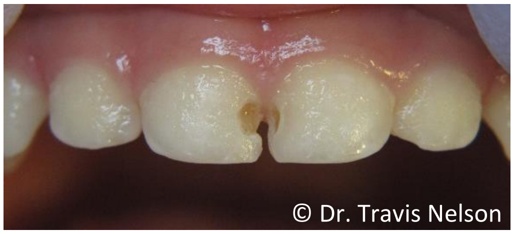

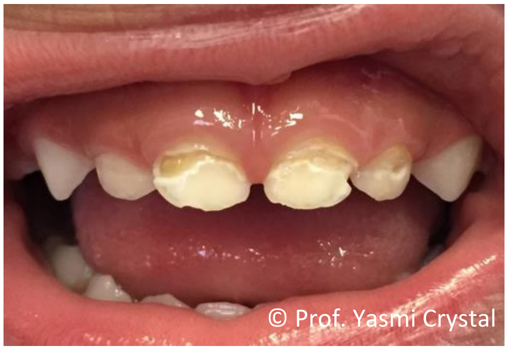

After SDF treatment

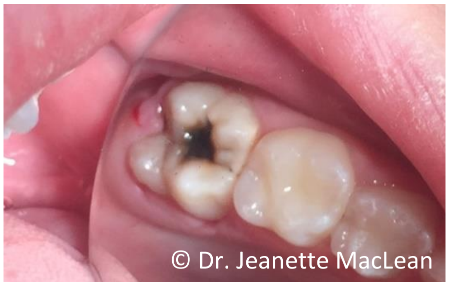

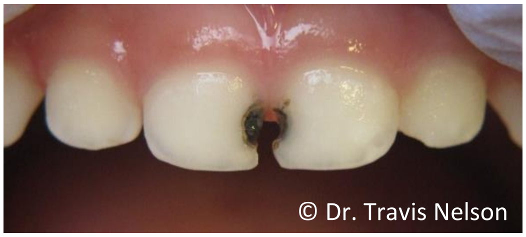

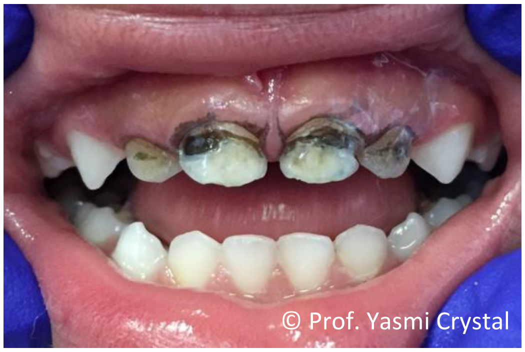

Supplement: Supplementary file 2 — Additional file 2. Photographs shown to parents and children as part of the interviews. [file 12903_2021_1730_MOESM2_ESM.pdf]
